# IMP1 is a novel posttranscriptional regulator of early erythroid development and embryonic globin output^∗^

**DOI:** 10.1182/bloodadvances.2025019406

**Published:** 2026-06-11

**Authors:** Katherine A. MacInnes, Ivan Ferrer-Vicens, Minna Hancock, Kongtana Trakarnsanga, Deborah E. Daniels, Marieangela C. Wilson, Jan Frayne

**Affiliations:** 1 School of Biochemistry, University of Bristol, Bristol, United Kingdom; 2 Department of Biochemistry, Faculty of Medicine Siriraj Hospital, Mahidol University, Bangkok, Thailand; 3 Proteomics Facility, Faculty of Health and Life Sciences, University of Bristol, Bristol, United Kingdom

## Abstract

The RNA-binding protein IMP1 is a developmentally expressed, posttranscriptional regulator involved in mRNA stability and translation. In the erythroid lineage, IMP1 was found to be expressed in fetal but repressed in adult erythroid cells, being among the most differentially expressed proteins between these developmental stages, with levels decreasing sequentially as erythroid ontogeny progresses. To investigate its role in early erythroid cells, IMP1 was knocked down in the fetal erythroid line HUDEP-1. Knockdown did not alter expansion or differentiation. However, tandem mass tag–based proteomics revealed significant changes in the proteome, correlating with differential abundance of proteins in fetal compared with adult erythroid cells, indicating a role in specification of the fetal erythroid phenotype. Notwithstanding, there was no significant effect of IMP1 knockdown or knockout in HUDEP-1 on fetal γ-globin levels. However, there was a marked, significant reduction in embryonic ζ- and ε-globin protein levels, which, along with a strong association of IMP1 with their transcripts, supports direct posttranscriptional regulation by IMP1. Furthermore, IMP1 was found to be significantly more abundant in human induced pluripotent stem cells (iPSC)-embryonic erythroid cell surrogates than even fetal erythroid cells, suggesting a potentially more predominant role at this earlier, poorly characterized stage of erythroid development. This study uncovers a novel role for IMP1, reveals a previously unrecognized regulatory mechanism in early erythroid cells, and highlights the significant contribution of epitranscriptomic regulation in embryonic globin expression. As reactivation of embryonic globins in definitive erythroid cells holds therapeutic promise for hemoglobinopathies, targeting the mechanisms by which IMP1 enhances embryonic globin translation could complement transcriptional strategies, enabling therapeutic levels to be achieved.

## Key Points

• Epitranscriptomic reader IMP1 is a novel regulator of fetal erythroid cell phenotype.
• Embryonic globin expression is directly regulated by IMP1 at the posttranscriptional level.

## Visual Abstract

**Figure:**
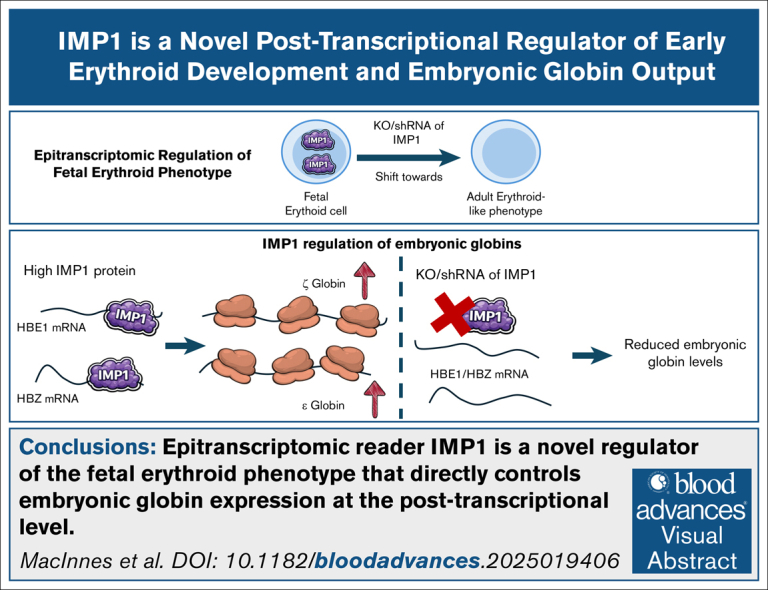

## Introduction

Insulin-like growth factor 2 (IGF-2) mRNA-binding protein 1 (*IGF2BP1*/IMP1) belongs to a conserved family of single-stranded RNA-binding proteins, along with IMP2 and IMP3.^1^ IMP1 is highly expressed during embryogenesis, where it plays a pivotal role in the regulation of developmental processes.^2^ Its expression is largely silent after birth but reactivated in certain adult cancers,^1^ thus being classified as an oncofetal protein.

Functioning as an interpreter of the epitranscriptome, IMP1 is an N6-methyladenosine (m6A) reader,^3^ translating RNA modification into functional consequence. It is thus a key regulator of posttranscriptional gene expression. IMP1 binding is primarily associated with stabilizing target mRNAs, thereby enhancing their translation.^3^ However, it has been shown to destabilize certain transcripts,^4^^,^^5^ leading to reduced protein production.

During erythroid ontogeny, the principal site of erythropoiesis changes, generating successive waves of erythroid cells with distinct phenotypes (reviewed by Palis),^6^^,^^7^ to meet the needs of the developing embryo. The initial transient wave occurs in the yolk sac, producing primitive embryonic erythroid cells. This is followed by the production of definitive erythroid cells in the fetal liver (FL) and, from around the time of birth, the bone marrow. However, the genetic programs underlying these developmental transitions are not well defined, with the regulation of human embryonic erythroid cells particularly poorly understood. The most distinctive difference between the different developmental stage erythroid cells is their globin expression profile, which transitions from predominant embryonic globin subunits ε- and ζ-globin in embryonic cells, to γ- and α-globin in fetal, and finally β- and α-globin in adult erythroid cells.

Consistent with its general developmental expression profile, IMP1 was found to be expressed in erythroid cells differentiated from umbilical cord blood (CB) but repressed in erythroid cells from adult peripheral blood (PB) hematopoietic stem cells (HSC).^8^ Ectopic overexpression of IMP1 in adult erythroid cells was shown to inhibit translation of γ-globin repressor BCL11A, leading to elevated γ-globin levels.^8^ However, the role of IMP1 in early erythroid cells, where it is intrinsically expressed, remains unexplored, pertinent given the growing recognition of posttranscriptional control as a key determinant of cellular homeostasis.^9^ Indeed, the importance of translational regulation in human erythroid cells is highlighted by the disparity between the erythroid cell transcriptome and proteome.^10^^,^^11^

In this study, we apply gene ablation strategies along with comparative proteomic analyses and RNA immunoprecipitation (RIP) to elucidate the function of IMP1 in early erythroid cells.

## Materials and methods

### Isolation and culture of CD34^+^ cells

Human FL CD34^+^ HSCs were isolated from FL donations provided by the Joint Medical Research Council/Wellcome Trust Human Developmental Biology Resource (grant MR/R006237/1; www.hdbr.org), with appropriate written consent and approval from the Newcastle and North Tyneside National Health Service Health Authority Joint Ethics Committee (18/NE/0290). Human Developmental Biology Resource is regulated by the UK Human Tissue Authority (www.hta.gov.uk) and operates in accordance with the relevant Human Tissue Authority Codes of Practice. CB units and leukocyte reduction system cones were provided with informed consent, used in accordance with the Declaration of Helsinki, and approved by the National Health Service National Research Ethics Committee (08/H0102/26) and the Bristol Research Ethics Committee (12/SW/0199). Adult PB and CB mononuclear cells were separated using Histopaque 1077. FL tissue was minced, digested with 1 mg/mL Collagenase IV, filtered, and washed in magnetic-activated cell sorting buffer. CD34^+^ cells were isolated using magnetic bead separation (Miltenyi Biotec) and differentiated using a 3-stage erythroid culture system.^12^

### Expansion and differentiation of HUDEP-1 cells

HUDEP-1 cells^13^ were cultured as described in the study by Daniels et al.^14^ In brief, cells were maintained in expansion medium (StemSpan SFEM [Stemcell Technologies] supplemented with 50 ng/mL stem cell factor (SCF), 3 U/mL erythropoietin (EPO), 1μM dexamethasone, and 1 μg/mL doxycycline). For differentiation, cells were transferred to differentiation medium (Iscove modified Dulbecco medium; Merck]; with 3% [volume-to-volume ratio] human AB serum, 2% [volume-to-volume ratio] fetal bovine serum, 10 μg/mL insulin, 3 U/mL heparin, 200 μg/mL holo-transferrin, 1 ng/mL interleukin-3, 3 U/mL EPO, 10 ng/mL SCF, 1 U/mL penicillin/streptomycin, and 1 μg/mL doxycycline) for 4 days, followed by a further 4 days without doxycycline. From day 8 onward, cells were cultured in differentiation medium without doxycycline, interleukin-3, or SCF, supplemented with an additional 300 μg/mL holo-transferrin.

### Lentiviral preparation and transduction

Lentiviral particles encoding IMP1 short hairpin RNA (shRNA; TRCN0000218799) or scrambled control were produced in Lenti-X HEK293T cells. Viral supernatants were harvested 48 hours after transfection and concentrated using Lenti-X Concentrator. For transduction, HUDEP-1 cells were incubated with concentrated virus for 24 hours, washed, recovered for 24 hours, and then selected with puromycin (1-2 μg/mL) for 48 hours before returning to expansion medium.

### CRISPR gene editing of HUDEP-1 cells

Single guide RNA (sgRNA) oligonucleotides (supplemental Table 1) were commercially synthesized by Synthego. HUDEP-1 cells were edited using Cas9-sgRNA ribonucleoprotein complexes. Ribonucleoproteins were prepared by incubating 18 pmol Cas9 with 45 pmol synthetic sgRNA (Synthego) for 15 minutes at 25°C. Cells (1 × 10^5^) were transfected using an Amaxa 4D-Nucleofector (DZ100 program) in P3 nucleofection (P3 NF) solution and recovered in expansion medium for 2 to 3 days. Live cells were single-cell sorted by fluorescence-activated cell sorting (DRAQ7 dye exclusion) into 96-well plates and expanded for 2 to 4 weeks. Clone screening was performed by polymerase chain reaction (PCR), purification, and Sanger sequencing, with insertions and deletions (indels) analyzed using Benchling and the Synthego ICE tool.

### PCR and quantitative reverse transcription–PCR analysis

Genomic DNA was extracted and IMP1 fragments amplified using GoTaq polymerase (Thermo Fisher Scientific). RNA was extracted, reverse transcribed to complementary DNA (cDNA), and quantitative PCR (qPCR) performed with Syber Select Master Mix on a QuantStudio 3, normalizing target gene expression to PABPC1 using the 2ˆ−ΔΔCt method. Primer sequences are provided in supplemental Table 2.

### Flow cytometry

For intracellular markers, cells were fixed (1% paraformaldehyde + 0.0075% glutaraldehyde), permeabilized (0.1% saponin), and antibodies added in 0.1% saponin with 4% phosphate-buffered saline–Bovine Serum Albumin and Glucose (AG). For surface markers, antibodies were added in 1% phosphate-buffered saline–AG. Nonviable cells were excluded with propidium iodide. Data were acquired on a BD LSRFortessa X-20 and analyzed with FlowJo.

### Antibodies

For western blotting, primary antibodies were as follows: IMP1 (clone 6A9) and IMP2 (Stefan Hüttelmaier); IMP3 (ab179807), BCL11A (ab19487), carbonic anhydrase 1 (CA1; ab6618), ε-globin (ab156041), ZBTB7A (ab175918), CDH1 (ab40772), TFRC (ab214039) all Abcam; β-globin (sc-21757), γ-globin (sc-21756), ARID3A (sc-398367), TFR2 (sc-32271) all Santa Cruz Biotechnology; ζ-globin (MAB7708; R&D Systems), β-actin (A1978; Sigma-Aldrich), MAPK1 (9102; Cell Signaling Technology). Horseradish peroxidase–conjugated secondary antibodies were from Dako.

For flow cytometry, Band 3 (BRIC71) and Glycophorin A (BRIC256) were from the IBGRL, Bristol, United Kingdom. Anti–α4 integrin-FITC (130-093-283) and anti–CD36-FITC (130-095-470) were from Miltenyi Biotec. APC-conjugated anti–mouse immunoglobulin G1 secondary antibody was from BioLegend.

### Reverse-phase HPLC for globin detection

Reverse-phase high-performance liquid chromatography (HPLC) was performed as previously described^15^ using a Phenomenex Jupiter C18 column equipped with a SecurityGuard cartridge using an UltiMate 3000 HPLC system.

### Comparative proteomics

Comparative proteomic analysis was performed as previously described.^11^ Whole-cell lysates were digested with trypsin, tandem mass tag (TMT) labeled (Thermo Fisher), and fractionated by high-pH reversed-phase chromatography. Peptide fractions were analyzed by nano–liquid chromatography (LC)/tandem mass spectrometry (MS/MS) on an Orbitrap Fusion Tribrid mass spectrometer (Thermo Fisher) operated in synchronous precursor selection (SPS)-mass spectrometry (MS)^3^ mode. MS data were processed and quantified using Proteome Discoverer (version 2.1) and searched against the human UniProt database, applying a 5% false discovery rate. Common contaminants and low-confidence identifications were excluded. Protein abundances were normalized to total peptide amount and log_2_ transformed. Statistical significance was determined using the Welch *t* test with Benjamini-Hochberg correction for multiple comparisons. Transcription factors among significantly altered proteins were identified by cross-referencing results with TFCheckpoint 2.0 and the curated human TF catalog.^16^

### RIP

HUDEP-1 cells were cross-linked with 0.1% paraformaldehyde and quenched with 0.125M glycine. Cells were lysed in RIP-lysis buffer supplemented with 2mM phenylmethylsulfonyl fluoride (PMSF), 1mM dithiothreitol (DTT), and RNaseOUT on ice for 30 minutes, treated with TURBO DNase, and clarified by centrifugation. Lysates were incubated with antibody-bound beads for 2 hours at 4°C, washed with RIP lysis buffer and high-salt buffer (RIP lysis buffer with 0.5M NaCl), and cross-links reversed using Proteinase K. RNA was extracted with TRIzol LS, precipitated with GlycoBlue, washed with 75% ethanol, DNase-treated, acidic phenol extracted, ethanol-precipitated overnight at −20°C, and resuspended for cDNA synthesis using SuperScript IV (Invitrogen).

### Statistical analysis

Comparisons between 2 groups were performed using unpaired 2-tailed *t* tests with Welch correction. For comparisons of >2 groups, ordinary 1-way analysis of variance with Tukey multiple comparisons test was used. Multiple unpaired *t* tests were applied for specific pairwise comparisons. Correlations were assessed using Pearson *r*. All analyses were performed in GraphPad Prism 10.6.0.

## Results

### Dynamic expression of IMP1 during erythroid ontogeny

**Figure 1.**
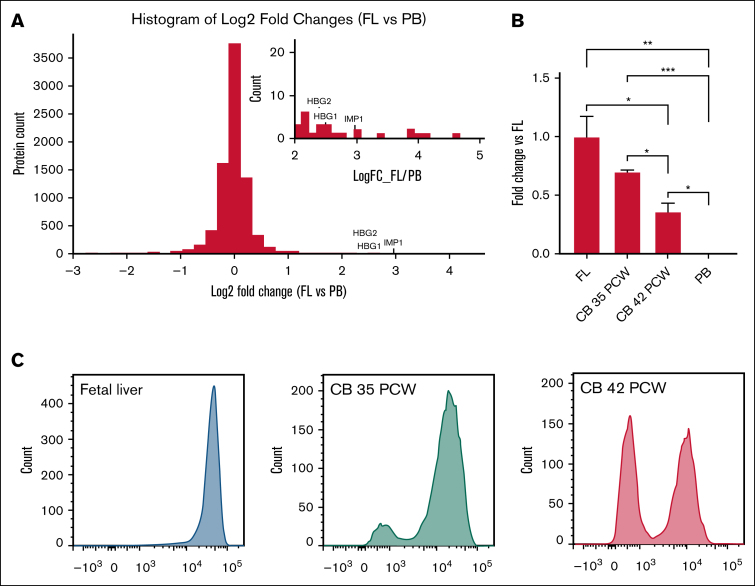
**IMP1 is highly expressed in fetal erythroid cells and decreases as erythroid ontogeny progresses.** (A) Histogram of log_2_ fold difference in protein levels between erythroid cells differentiated from FL and adult PB CD34^+^ cells analyzed by TMT-based proteomics. Inset highlights proteins with the greatest differential levels; IMP1 and γ-globin (HBG1/2) are indicated. (B) Densitometric analysis of IMP1 protein abundance from western blot analysis (representative western shown in supplemental Figure 1), normalized to β-actin. Data represent mean ± standard error of the mean (SEM) for erythroid cells differentiated from FL, CB (35 and 42 PCW), and PB CD34^+^ cells. (C) Representative flow cytometry histograms of IMP1 levels in erythroid cells differentiated from FL, 35 and 42 PCW CB. All data are from erythroid cells differentiated for 7 days to induce the erythroid transcriptional program and globin expression. Statistical significance was assessed using 1-way analysis of variance (ANOVA) with multiple comparisons. ∗*P* < .05; ∗∗*P* < .01; ∗∗∗*P* < .001.

To identify molecular regulators of erythroid ontogeny, TMT-based proteomics was performed on erythroid cells differentiated from human FL and adult PB CD34^+^ HSCs. The data showed γ- and β-globin among the topmost differentially expressed proteins, higher in fetal and adult cells, respectively, as expected, validating the approach. RNA-binding protein IMP1 was also among the most differentially expressed proteins (Figure 1A). Data were corroborated by orthogonal methods with erythroid cells differentiated from FL at 10 to 17 postconception weeks (PCW), CB 35 PCW, CB 42 PCW, and adult PB CD34^+^ cells (Figure 1B-C; supplemental Figure 1), also revealing a progressive decrease in IMP1 levels in erythroid cells through development; IMP1 was significantly higher in FL than at all other developmental stages. The increasing proportion of IMP1^low^ cells in 35 PCW and 42 PCW CB samples (Figure 1C) was consistent with the developmental transition toward the adult erythropoietic program. Abundance of IMP1 was also higher in earlier (FL 10 and 11 PCW) than later FL erythroid cells (supplemental Figure 1). The data suggest IMP1 is a posttranscriptional regulator of developmental stage–specific erythroid cells.

### Loss of IMP1 does not impair erythroid cell differentiation

**Figure 2.**
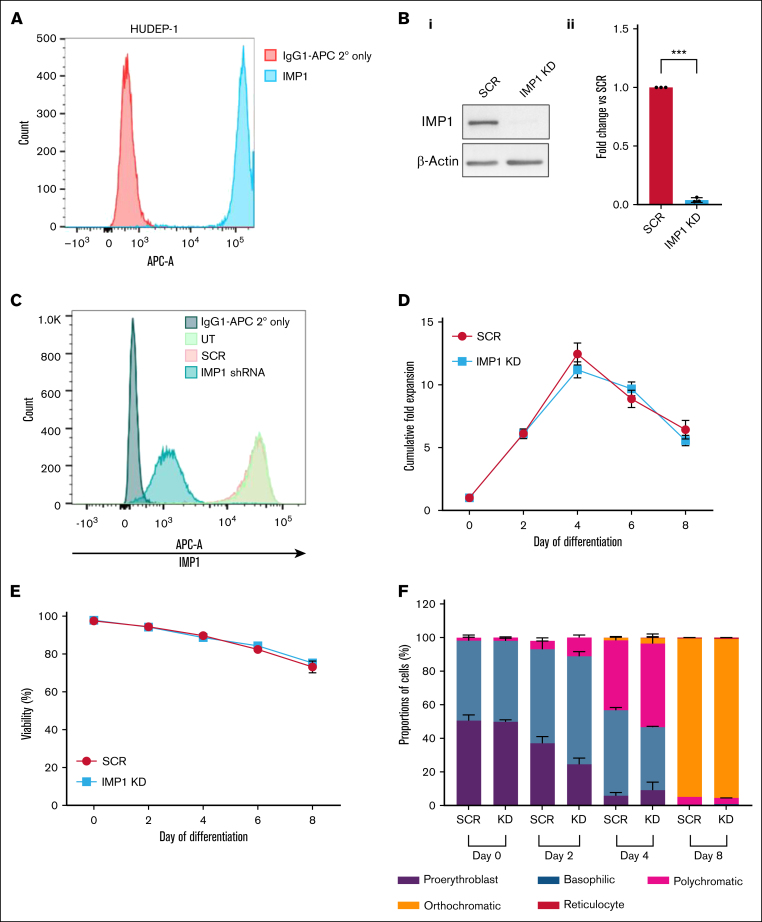
**IMP1 knockdown in HUDEP-1 cells does not impair erythroid differentiation.** (A) Representative flow cytometry histogram of IMP1 (APC) levels in expanding HUDEP-1 cells. (Bi) Representative western blot of IMP1 protein levels in cells transduced with IMP1 shRNA (IMP1 KD) vs scrambled (SCR) shRNA control, with (Bii) quantification of IMP1 knockdown efficiency from 3 independent replicates, normalized to β-actin. (C) Flow cytometry histogram of IMP1 (APC) levels in IMP1 shRNA compared with control SCR shRNA transduced and untransduced (UT) cells. (D) Cumulative fold expansion and (E) cell viability during erythroid differentiation of IMP1 KD and control (SCR) cells. (F) Morphological analysis from Leishman Eosin-Methylene Blue–stained cytospins of IMP1 shRNA (KD) and control SCR shRNA transduced cells at days 0, 2, 4, and 8 of culture. At least 200 cells per slide were scored. Mean ± SEM, n = 3, ∗∗∗*P* < .001.

To investigate its role in fetal erythroid cells, IMP1 was knocked down in HUDEP-1, a fetal erythroid cell line that expresses predominantly fetal hemoglobin (HbF)^13^ commensurate with primary FL erythroid cells. Endogenous IMP1 protein level in HUDEP-1 is also consistent with that of fetal erythroid cells (Figure 2A). Stable knockdown (KD) of IMP1 was achieved using lentiviral delivery of IMP1 shRNA. Western blot and flow cytometry confirmed near-complete loss of IMP1 in IMP1 shRNA-transduced cells, with no change in the scrambled shRNA-transduced control compared with untransduced cells (Figure 2Bi-C). Knockdown efficiency was reproducible across 3 independent replicates (Figure 2Bii). Following knockdown, HUDEP-1 cells were differentiated using a 3-stage erythroid culture system.^14^ IMP1 KD and control cells showed comparable expansion and viability throughout differentiation (Figure 2D-E). Morphological analysis at days 0, 2, 4, and 8 of differentiation demonstrated consistent maturation kinetics between IMP1 KD and control cells (Figure 2F; supplemental Figure 2A), confirmed by flow cytometry of established membrane markers for erythroid differentiation^14^ (supplemental Figure 2B-C). The data show that IMP1 knockdown does not disrupt erythroid cell proliferation or differentiation.

### Remodeling of fetal erythroid proteome following IMP1 depletion

**Figure 3.**
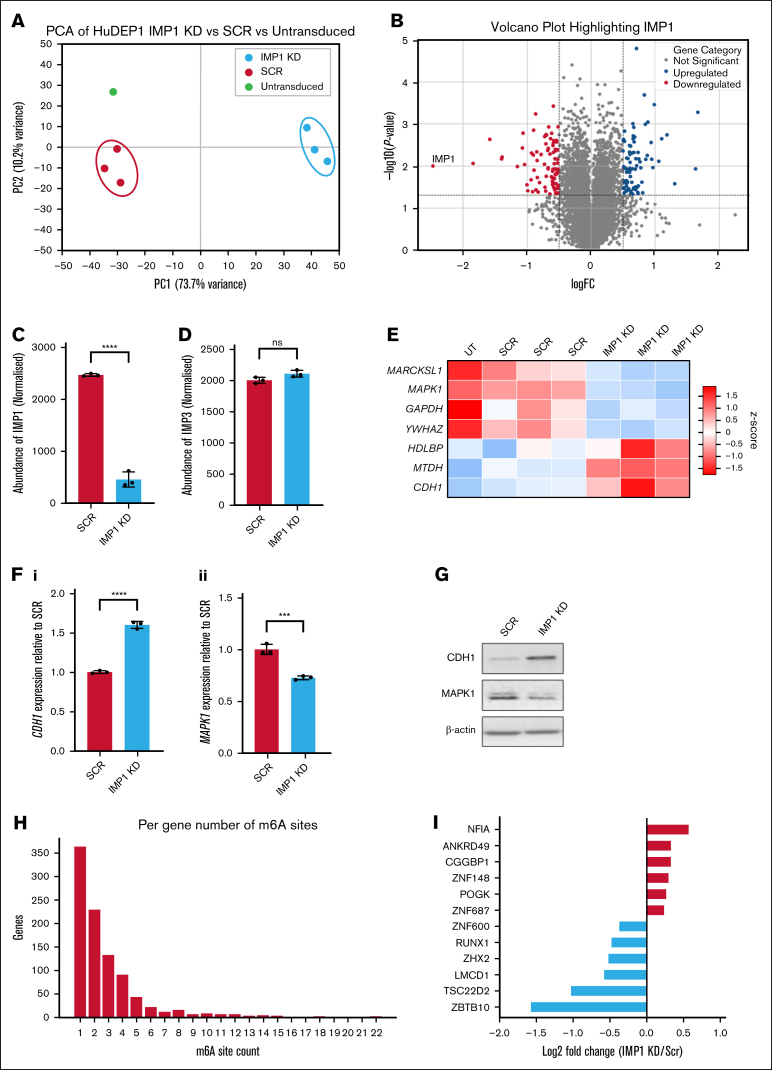
**Remodeling of the erythroid proteome following IMP1 depletion.** (A) Principal component analysis of TMT-based quantitative proteomics showing separation between the proteome of IMP1 shRNA (IMP1 KD) and control shRNA (SCR) transduced and UT HUDEP-1 cells. (B) Volcano plot displaying significantly upregulated (blue) and downregulated (red) proteins in IMP1 KD relative to control HUDEP-1 cells; IMP1 is indicated. (C) Scaled abundance of IMP1 and (D) IMP3 protein in control (SCR) vs IMP1 KD HUDEP-1 cells. (E) Heat map illustrating significantly altered levels of previously validated IMP1 targets in IMP1 KD compared with control (SCR) and UT HUDEP-1 cells. (F) Transcript abundance analyzed by qRT-PCR, and (G) protein abundance of CHD1 and MAPK1 in IMP1 KD vs control (SCD) HUDEP-1 cells; β-actin was used as a protein loading control. (H) Proportion of differentially expressed proteins in IMP1 KD HUDEP-1 cells whose transcripts carry known m^6^A modifications (as reported in human erythroleukemia cells). (I) Log_2_ fold change of the top 6 upregulated and top 6 downregulated transcription factors significantly altered in the proteomic data set following IMP1 KD; upregulated factors are shown in red, downregulated in blue. Mean ± SEM, n = 3, ∗∗∗*P* < .001; ∗∗∗∗*P* < .0001.

To determine the role of IMP1 in erythroid cells, TMT-based quantitative proteomics was next performed on HUDEP-1 IMP1 KD and control cells. A total of 7388 unique proteins were quantified (supplemental Table 3). Principal component analysis showed clear separation between IMP1 KD and control cells along principal component 1, which represents 73.7% of the variance, indicating a strong and reproducible shift in the proteome (Figure 3A), also illustrated by a volcano plot (Figure 3B). Of the 7388 proteins, 1758 (∼24%) were significantly altered in level, 967 (13%) decreased, and 791 (∼11%) increased in IMP1 KD cells compared with controls. IMP1 was the most downregulated protein, further confirming effective KD (Figure 3B-C). The level of paralog IMP3 remained unchanged following IMP1 depletion, indicating no compensatory upregulation (Figure 3D). Of the 21 previously confirmed IMP1 targets^17^^,^^18^ identified in the data set, 7 were significantly altered in level following IMP1 KD (Figure 3E), validating the approach, with a lack of change in others indicating cell type–specific regulation by IMP1. Two of these targets were further validated, with transcript and protein levels consistent with the proteomic data (Figure 3F-G). In line with the unaffected differentiation kinetics of IMP1 KD cells, the abundance of key erythroid transcription factors KLF1 and GATA1 was unchanged (supplemental Figure 3).

To provide insights into whether the mRNA for proteins that change in level on IMP1 KD may be direct IMP1 targets, we cross-referenced the IMP1 KD proteomic data with a data set of transcripts with confirmed m6A sites in human erythroleukemia cells.^19^ Of the proteins significantly altered in level on IMP1 knockdown, 932 correspond to transcripts with at least 1 m6A modification (Figure 3H), with most m6A marks located near the stop codon or within the 3′ untranslated region (UTR) where IMP1 predominantly binds,^19^ suggesting potential direct posttranscriptional regulation of a large number of the proteins by IMP1. Other changes in the proteome are no doubt because of indirect/downstream effects, potentially caused at least in part by the altered abundance of some transcription factors on IMP1 KD (Figure 3I, top 6 increased and decreased shown).

### IMP1 contributes to the maintenance of fetal erythroid cell phenotype

**Figure 4.**
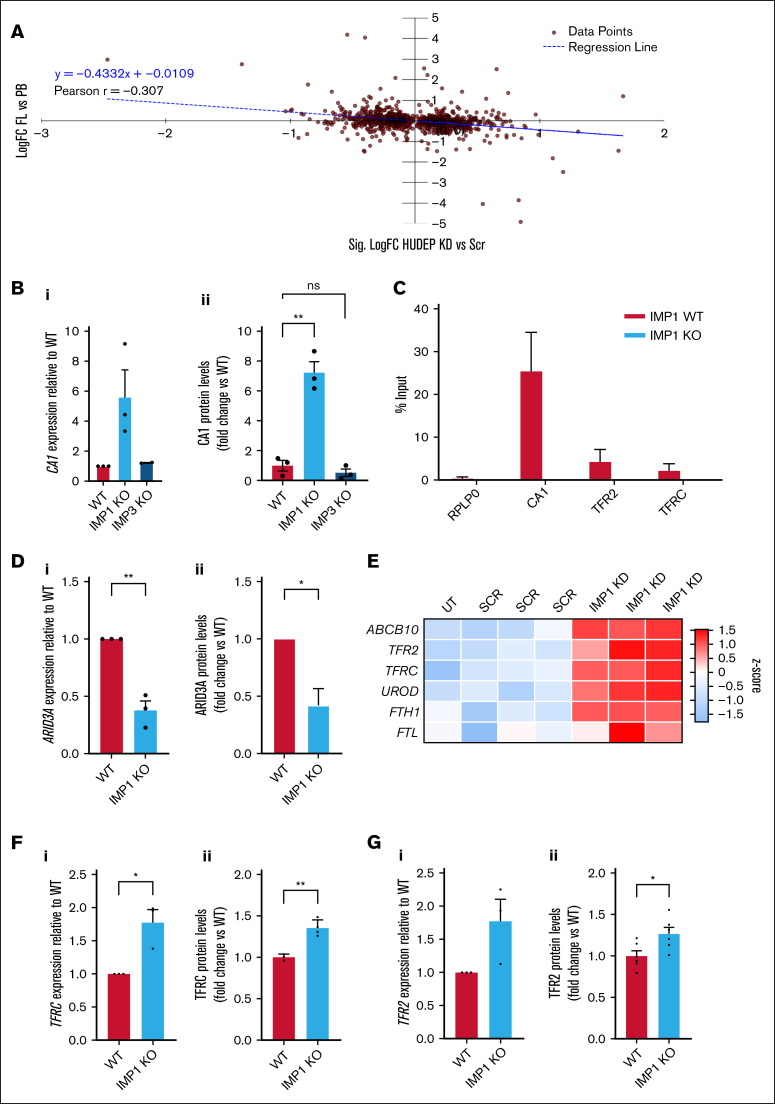
**IMP1 is involved in the specification of fetal erythroid phenotype.** (A) Correlation analysis between proteins significantly altered in level in the IMP1 KD HUDEP-1 proteome and an in-house fetal vs adult erythroid cell proteomic data set at the same stage of differentiation. Pearson correlation coefficient (*r*) is shown. The regression line is represented as a blue dotted line. (Bi) *CA1* transcript levels in control UT pool wild-type (WT), IMP1 KO, and IMP3 KO HUDEP-1 cells analyzed by qRT-PCR. (Bii) Densitometric analysis of CA1 protein abundance from western blot analysis (representative western shown in supplemental Figure 6B), normalized to β-actin. (C) RIP followed by qPCR (RIP-qPCR) in HUDEP-1 cells showing enrichment of *CA1*, *TFR2*, and *TFRC* transcripts with IMP1 antibody. Immunoglobulin G control, IMP1 KO cells, and non-IMP1 target transcript *RPLP0* were included to confirm specificity. Data are presented as a percentage of input from ≥2 independent experiments. (Di) *ARID3A* transcript levels in WT and IMP1 KO cells measured by qRT-PCR. (Dii) Densitometric analysis of ARID3A protein abundance from western blot analysis (representative western shown in supplemental Figure 6C), normalized to β-actin. (E) Heat map of z scored abundance values for iron metabolism–associated proteins in IMP1 shRNA (IMP1 KD) and control shRNA transduced (SCR), and UT HUDEP-1 cells. (Fi) qRT-PCR analysis of *TFRC* in control UT pool (WT) and IMP1 HUDEP-1 KO cells. (Fii) Densitometric analysis of TFRC protein abundance from western blot analysis (representative western shown in supplemental Figure 6F), normalized to β-actin. (Gi) qRT-PCR analysis of *TFR2* in control (WT) and IMP1 HUDEP-1 KO cells. (Gii) Densitometric analysis of TFR2 protein abundance from western blot analysis (representative western shown in supplemental Figure 6G) normalized to β-actin. Data are presented as mean ± SEM. ∗*P* < .05; ∗∗*P* < .01. ns, not significant.

To determine if IMP1 is required for developmental stage specification during erythroid ontogeny, the differential proteome of IMP1 KD HUDEP-1 cells was compared with a comparative proteomic data set of human fetal vs adult erythroid cells at the same stage of differentiation (3 biological repeats of each; K.A. MacInnes, M.C. Wilson and J. Frayne, unpublished data, June 2026). A significant negative correlation was obtained (Pearson *r* = −0.305; Figure 4A), indicating IMP1 depletion caused transition of the proteome to a more adult-like state, thus supporting a role for IMP1 in maintenance of the fetal erythroid phenotype.

To further validate the IMP1 KD findings, IMP1 knockout (KO) HUDEP-1 cell lines were generated using CRISPR gene editing (supplemental Figure 4). The differentiation kinetics of IMP1 KO lines were unchanged (supplemental Figure 5), as found for the IMP1 KD lines. In addition, HUDEP-1 IMP3, a paralog with a similar developmental expression profile, KO lines (supplemental Figures 1 and 4) were created to confirm the specificity of observed effects to IMP1.

Looking more closely at developmental markers, CA1, normally present in adult but not fetal erythroid cells^20^ (supplemental Figure 6A), was significantly increased in IMP1 KD HUDEP-1 cells (supplemental Table 3), with both transcript and protein abundance also increased in IMP1 KO cells (Figure 4B; supplemental Figure 6B). These data correlate with the converse depletion of CA1 on overexpression of IMP1 in adult erythroid cells.^8^ Furthermore, IMP1 bound strongly to *CA1* mRNA in the IMP1 RIP-qPCR assay (Figure 4C), supporting direct posttranscriptional regulation. Neither the CA1 transcript nor the protein level was altered in IMP3 KO cells (Figure 4B; supplemental Figure 6B). In contrast, the embryonic/fetal erythroid cell marker ARID3A^21^ (also see Figure 6B) was significantly reduced in level in the IMP1 KD proteomic data set, with transcript and protein abundance also significantly decreased in the IMP1 KO cells (Figure 4D; supplemental Figure 6C). IMP1 has been shown to bind *ARID3A* mRNA in human embryonic stem cells (hESC),^17^ again suggesting direct regulation.

Several iron-related proteins were significantly upregulated on IMP1 KD (Figure 4E), with validation of data for transferrin receptors TFRC and TFR2 (supplemental Figure 6D-E), also in IMP1 KO cells, confirming increased levels of transcript and protein (Figure 4F-G; supplemental Figure 6F-G). Additionally, RIP-qPCR revealed that IMP1 binds to *TFRC* and *TFR2* transcripts (Figure 4C), although the enrichment was lower than that observed for CA1, consistent with the more modest change in TFRC and TFR2 protein levels, and suggesting a subtler regulatory role.

### IMP1 is a novel posttranscriptional regulator of embryonic ζ- and ε-globins

Ectopic overexpression of IMP1 in adult erythroid cells was previously shown to impede translation of *BCL11A* mRNA, reducing BCL11A protein level and thus the repression of γ-globin transcription,^8^ in part because of increased interaction of the γ-globin gene with the locus control region,^22^ resulting in increased γ-globin.

**Figure 5.**
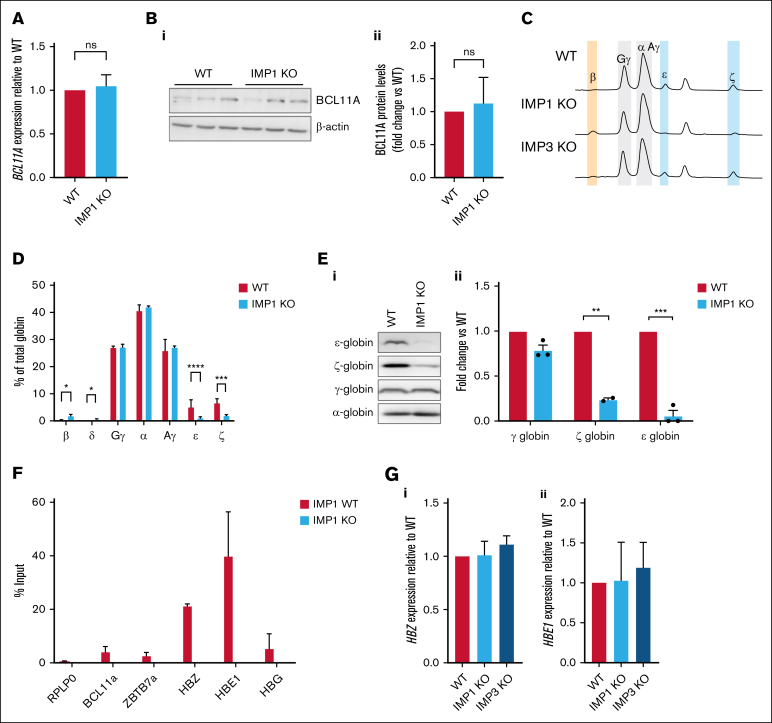
**IMP1 KO reduces embryonic globin levels.** (A) *BCL11A* transcript levels measured by qRT-PCR in control UT pool (WT) and IMP1 KO HUDEP-1 cells. (Bi) Western blot of BCL11A in WT and IMP1 KO cells, with (Bii) densitometric analysis of the BCL11A protein levels normalized to β-actin. (C) Representative RP-HPLC chromatograms comparing globin subunit levels in WT, IMP1 KO, and IMP3 KO HUDEP-1 cells on day 8 of differentiation, with (D) quantification of RP-HPLC peak areas from 3 independent IMP1 KO clones. (Ei) Representative western blot of globin subunit levels in IMP1 KO compared with WT HUDEP-1 cells, with (Eii) densitometric quantification of the globin levels from 3 independent IMP1 KO clones, normalized to α-globin. (F) RIP-qPCR showing enrichment of *BCL11A*, *ZBTB7a*, *HBE1* (ε-globin), *HBZ* (ζ-globin), and *HBG* (γ-globin) transcripts using IMP1 antibody in WT HUDEP-1 cells. No enrichment was observed with immunoglobulin G control, IMP1 KO cells, or for the negative control transcript *RPLP0*. (Gi-ii) qRT-PCR analysis of *HBZ* and *HBE1* in control UT pool (WT), IMP1 KO, and IMP3 KO cells. Data are presented as mean ± SEM. ∗*P* < .05; ∗∗*P* < .01; ∗∗∗*P* < .001; ∗∗∗∗*P* < .0001. ns, not significant.

However, there was no significant effect of IMP1 KO on the abundance of *BCL11A* transcripts (Figure 5A) or protein (Figure 5B) in HUDEP-1 cells and no significant change in the level of γ-globin on reversed-phase (RP)-HPLC analysis or western blot on IMP1 KO (Figure 5C-E) or KD (supplemental Figure 7A-D). There was also no change in transcript or protein level of another established γ-globin repressor, ZBTB7A^23^ (supplemental Figure 8A-B).

A slight increase in key adult globins β-, δ-, and α-globins was observed on IMP1 KO and KD in HUDEP-1 cells, in some instances reaching significance (Figure 5C-D; supplemental Figure 7C-D), correlating with the shift to a more adult erythroid phenotype.

However, there was a striking, significant decrease in abundance of embryonic ζ- and ε-globin protein on IMP1 KO and KD (Figure 5C-E; supplemental Figure 7A-D), the main globins of embryonic erythroid cells, but still expressed, although at lower levels, in fetal cells. Furthermore, RIP-qPCR analysis in control HUDEP-1 cells revealed strong enrichment of ζ- and ε-globin transcripts by IMP1 (Figure 5F), revealing novel posttranscriptional regulation of these globins by IMP1 but with unchanged level of their transcripts on IMP1 depletion (Figure 5G; supplemental Figure 7H). Weak binding was also observed for *BCL11A*, *ZBTB7A*, and *γ-globin* mRNA (Figure 5F) but with no significant detectable functional outcomes. No enrichment was observed in IMP1 KO cells or with immunoglobulin G controls, confirming the specificity of the interactions. There was no effect of IMP3 KO or double IMP1 plus IMP3 KO, beyond the effect of IMP1 depletion alone, on the abundance of any globins (Figure 5C; supplemental Figure 7E-G). Finally, both *HBE1* and *HBZ* were found to harbor the DRACH consensus motifs associated with m6A sites, with *HBE1* notably containing the canonical GGACU sequence (supplemental Figure 9). Although direct m6A modification remains to be experimentally verified, these sequence features support IMP1 regulation of embryonic ζ- and ε-globin transcripts.

### Validation of iPSC-derived erythroid cells as human embryonic erythroid cell surrogates reveals highly elevated levels of IMP1

**Figure 6.**
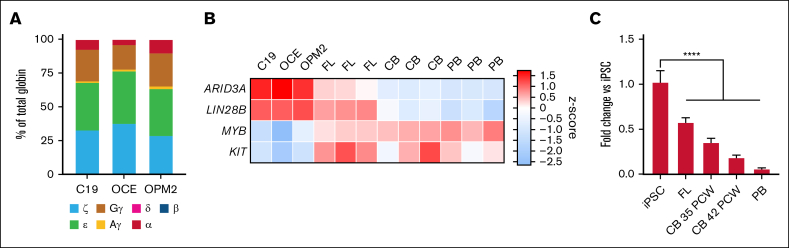
**IMP1 is highly associated with embryonic erythroid identity.** (A) Globin subunit composition in erythroid cells differentiated from 3 iPSC lines (C19, OCE, and OPM2) at day 8 in erythroid differentiation culture analyzed by TMT-based proteomics.^24^ (B) Heat map illustrating z scores of protein abundance of erythroid developmental stage–specific markers in erythroid cells differentiated from iPSC (C19, OCE, and OPM2)-derived, FL, CB, and PB CD34^+^ cells. (C) Relative IMP1 protein levels from pooled iPSC (C19, OCE, and OPM2), FL, CB, and PB-derived erythroid cells. Data are presented as mean ± SEM. ∗∗∗∗*P* < .0001.

Posttranscriptional regulation of embryonic globins by IMP1 suggests it may have a more predominant role in embryonic erythroid cells. As studying human embryonic erythroid cells is severely impeded by difficulty obtaining very early yolk sac tissue, we validated erythroid cells differentiated from iPSCs (C19, OCE, and OPM2 lines^24^) as models for human embryonic erythroid cells, as previously endorsed. The iPSC-derived erythroid cells expressed predominantly embryonic ζ- and ε-globin (Figure 6A). They also had significantly increased levels of embryonic markers ARID3A and LIN28B, and significantly lower levels of definitive erythroid cell markers Myb and KIT (Figure 6B) compared with all definitive erythroid cell populations (FL, CB 35, 42 PCW, and adult), as found for human embryonic erythroid cells (reviewed by Palis).^7^ The data validate the iPSC-derived cells as surrogates for embryonic erythroid cells. The abundance of IMP1 was found to be strikingly, significantly higher in the iPSC-derived than in fetal erythroid cells, as well as significantly higher than in CB and adult erythroid cells (Figure 6C), providing compelling evidence for a potentially more predominant role at this developmental stage.

## Discussion

In this study, we reveal the novel role of RNA-binding protein IMP1 in the erythroid lineage. At the fetal stage, it acts as an ontogenic regulator that contributes to the specification of a fetal erythroid phenotype, whereby IMP1 depletion caused progression to a more adult-like state. Notwithstanding, the loss of IMP1 did not affect fetal γ-globin levels but instead resulted in a marked reduction in embryonic ζ- and ε-globin. The strong interaction of IMP1 with ζ- and ε-globin transcripts suggests direct epitranscriptomic regulation by IMP1.

IMP1 was also significantly higher in human iPSC-embryonic erythroid cell surrogates, where ζ- and ε-are the predominant globins, than even early fetal erythroid cells, suggesting a role for IMP1 also at this earlier, poorly characterized stage of erythroid development, which is potentially more predominant than in fetal cells. This early prominence may diminish as erythroid ontogeny progresses, consistent with the observed sequential decline in IMP1 levels.

In addition, although IMP1 has been reported in the literature predominantly as a positive posttranscriptional regulator,^3^ our findings reveal both positive and negative regulatory effects. Transcripts for proteins that were increased as well as decreased on IMP1 KD in HUDEP-1 cells were shown in our RIP-qPCR assays to bind IMP1 and/or to have IMP1-binding sites in human erythroleukemia cells.^19^ Such dual effects may be attributed to the location of RNA-binding protein in the transcript^25^ but are also likely mediated by recruitment of cofactors, with some IMP1 cofactors reported, including mRNA stabilizers and inhibitors/degraders.^3^^,^^25^^,^^26^

Furthermore, the change in transcript abundance for IMP1 targets ARID3A, MAPK1, CA1, CDH1, TFR2, and TFRC paralleled the change in respective protein abundance. Decrease in both transcript and protein on IMP1 depletion suggests IMP1 normally acts to stabilize these targets. However, further experimentation is required to determine whether IMP1 regulates translational efficiency as well as mRNA stability. An increase in transcript and protein abundance on IMP1 depletion suggests IMP1 normally acts to promote the degradation of these targets, further supporting the dual function of IMP1.

Conversely, abundance of both *HBZ* and *HBE1* mRNA was largely unchanged upon IMP1 depletion, suggesting IMP1 increases their translation efficiency, a role reported for other gene transcripts.^27^, ^28^, ^29^ However, the effect on *HBZ* and *HBE1* mRNA stability may be masked by the inherent high stability of globin mRNAs.

Finally, the strong positive correlation (*r* = 0.95; *P* < .001) between IMP1-binding enrichment and the magnitude of protein-level change upon IMP1 depletion (supplemental Figure 10) suggests that transcripts more strongly bound by IMP1 are subject to tighter regulation.

Studies on differential expression of globin genes during ontogeny have focused primarily on transcriptional repression, largely of γ-globin^30^^,^^31^ because of its therapeutic relevance for β-hemoglobinopathies. In contrast, regulation of human embryonic *HBZ* and *HBE1* expression remains poorly understood, although key repressors of *HBG* expression, BCL11A, and ZBTB7A, have also been implicated in repression of *HBZ* and *HBE1* expression.^32^^,^^33^

In contrast, posttranscriptional regulation of globins remains largely unexplored. RNA-binding protein PUM1 was shown to interact with *HBG1* transcripts in adult erythroid cells, reducing translational efficiency and thereby decreasing Aγ-globin protein levels.^34^ Conversely, our data show IMP1 functions as a positive posttranscriptional regulator and of ζ- and ε-globin, with direct binding to their transcripts supporting a mechanistic role in enhancing their translation. Moreover, the near-complete loss of ζ- and ε-globin proteins following IMP1 depletion highlights the significant role of IMP1-mediated posttranscriptional regulation in embryonic globin expression.

In contrast to ectopic overexpression of IMP1 in adult erythroid cells, where IMP1 repressed translation of the γ-globin gene repressor BCL11A, thereby increasing γ-globin protein levels,^8^ IMP1 depletion in the fetal HUDEP-1 erythroid cells had no significant effect on BCL11A or γ-globin.

This may reflect the predominantly transcriptional regulation of BCL11A during ontogeny, with fetal cells expressing very low *BCL11A* transcript levels,^35^ such that any impact of IMP1 loss is negligible. Although we detected weak binding of IMP1 to BCL11A transcripts, this had no measurable outcome on BCL11A or γ-globin protein levels. Alternatively, BCL11A has been reported to be primarily regulated at the level of mRNA translation in fetal erythroid cells, whereby developmentally regulated RNA-binding protein LIN28B binds to and represses *BCL11A* mRNA translation.^36^ Thus, in adult erythroid cells, where LIN28B expression is silenced,^36^ overexpressed IMP1 may bind ectopically or redundantly to *BCL11A* transcripts, repressing their translation, but has no impact on BCL11A in fetal cells where LIN28B is expressed. Either mechanism would explain the lack of effect of IMP1 KD in the fetal cells on BCL11A and γ-globin levels. However, disruption of LIN28B binding sites in *BCL11A* cDNA was reported to increase their translation in transfected 293T cells,^36^ where IMP1 is expressed,^3^ suggesting IMP1 does not act redundantly with LIN28B.

In addition to impeded direct posttranscriptional regulation by IMP1, changes in the proteome of IMP1-depleted HUDEP-1 cells will include downstream effects, some mediated by alterations in the abundance of regulatory factors. Among these, 3 of the 6 transcription factors most increased in level on IMP1 KD (NFIA, CGGBP1, and ZNF687) are naturally at a significantly higher level in adult than fetal erythroid cells, in line with and potentially contributing to the shift to a more adult-like erythroid phenotype. Furthermore, considering the slight increase in certain globins upon IMP1 depletion, ZNF148 and NFIA are also significantly increased, with ZNF148 shown to activate globin gene expression broadly,^37^ and NFIA implicated in the switch from fetal-to-adult globin expression.^38^

Finally, reactivation of embryonic globins in definitive erythroid cells holds therapeutic promise for disorders caused by mutations in their adult paralogs: ε-globin as an alternative or synergistic approach to γ-globin induction for β-hemoglobinopathies and ζ-globin as the most promising therapeutic approach for α-thalassemia. Targeting the mechanisms by which IMP1 enhances embryonic globin translation could therefore complement transcriptional strategies to achieve therapeutic globin levels.

Conflict-of-interest disclosure: The authors declare no competing financial interests.
