# Supplementary material for: IMP1 is a novel posttranscriptional regulator of early erythroid development and embryonic globin output
Source: Blood Adv. 2026 Jun 11;10(16):5518–30. doi: 10.1182/bloodadvances.2025019406 (PMC13486811; doi:10.1182/bloodadvances.2025019406)
Supplement: Supplementary file 1 [file BLOODA_ADV-2025-019406-mmc1.pdf]

**IMP1 is a novel Post-Transcriptional Regulator of Early Erythroid Development and Embryonic Globin output**

**Supplementary Figure 1. IMP1 protein levels across developmental erythroid stages.**
Western blot of IMP1 levels in erythroid cells differentiated from Fetal liver (FL), Cord Blood (CB) post conception week (PCW) 35 and 42, and adult peripheral blood (PB) $CD34^+$ cells on day 7 of differentiation culture. β-actin was used as a protein loading control.

**Supplementary Figure 2. Characterization of IMP1 knockdown in HUDEP-1 cells.**
**(A)** May-Grünwald Giemsa–stained cytospins of IMP1 shRNA and control SCR shRNA transduced HUDEP-1 cells at days 0, 4, and 8 of differentiation. **(B)** Flow cytometry analysis of erythroid surface markers (CD36 with GPA and α4-integrin with Band3) during differentiation of IMP1 shRNA and control SCR shRNA transduced HUDEP-1 cells.

**Supplementary Figure 3. Generation and validation of IMP1 and IMP3 knockout HUDEP-1 clones.**
**(A)** Schematic of CRISPR-Cas9 targeting strategy for *IGF2BP1* (IMP1) and *IGF2BP3* (IMP3) gene editing. Coloured boxes represent coding exons. Guide RNA (gRNA) target sites used for CRISPR/Cas9 editing are indicated by black lines pointing to exon regions. The gRNAs target early coding exons to induce frameshift mutations. The PAM sequences are highlighted in red. **(B)** Western blot of IMP1 protein in three independent IMP1 KO clones. **(C)** Western blot of IMP3 protein in three independent IMP3 KO clones. Control (WT) are untransduced pool HUDEP-1 cells.

**Supplementary Figure 4. Morphological characterisation of IMP1 HUDEP-1 KO cells during erythroid differentiation.**
(**A**) Morphological analysis of three independent IMP1 KO and control untransduced pool (WT) HUDEP-1 cells from May-Grünwald Giemsa–stained cytospins at days 0, 2, 4, 6, 8, and 10 of erythroid differentiation. At least 200 cells per slide were scored.

**Supplementary Figure 5. Increase in CA1 protein during erythroid ontogeny, and TFR2 and TFRC transcripts on IMP1 KO.**
(**A**) Densitometric analysis of CA1 protein abundance from Western blots, normalized to β-Actin. Data are presented as mean ± SEM from FL, CB (35 and 42 post-conception weeks) and PB erythroid cells, n=3. (**B–C**) qRT-PCR analysis of TFR2 and TFRC in control untransduced pool (WT) and IMP1 HUDEP-1 KO cells. Data are presented as mean ± SEM. Statistical significance was determined by unpaired t-test. $P < 0.05$ (*), $P < 0.01$ (**).

**Supplementary Figure 6. Globin subunit abundance in IMP1 KD and IMP3 KO HUDEP-1 Cells.**
(**A**) Western blot of globin subunits in IMP1 shRNA (IMP1 KD) and control shRNAs (SCR) transduced HUDEP-1 cells at day 8 of erythroid differentiation, with (**B**) densitometric quantification of γ-, ζ- and ε-globin levels normalized to α-globin. (**C**) Representative reversed-phase high-performance liquid chromatography (RP-HPLC) chromatograms of globin subunit levels in IMP1 KD and control HUDEP-1 (SCR) cells on day 8 of differentiation, with (**D**) quantification of the HPLC peak area for each globin subunit, n=3. (**E**) Western blot of globin subunits in IMP3 KO compared to control untransduced pool (WT) HUDEP-1 cells, with (**F**) densitometric quantification of the globin levels from the three independent IMP3 KO clones, normalized to α-globin. (**G**) Representative RP-HPLC chromatograms of globin subunit levels in

WT, IMP1 KO, IMP3 KO, and double IMP1/IMP3 KO HUDEP-1 cells at day 8 of erythroid differentiation.

**Supplementary Figure 7. Analyses of *ZBTB7A* expression in WT and IMP1 KO HUDEP-1 cells.** (**A**) qRT-PCR analysis of *ZBTB7A* transcript levels in control untransduced pool (WT) and IMP1 KO HUDEP-1 cells. (**Bi**) Western blot of ZBTB7A protein levels in WT and IMP1 KO HUDEP-1 cells, with (**Bii**) densitometric quantification of the ZBTB7A protein levels, normalized to β-Actin; ns = not significant.

**Supplementary Figure 8. DRACH Motif Analysis in HBZ and HBE Genes.**
DRACH sites are indicated by vertical red lines. The canonical GGACU motif is highlighted with a vertical blue line.

**Supplementary Figure 9. Correlation between IMP1 enrichment and target protein-level on IMP1 depletion.** RIP-qPCR IMP1 enrichment (% Input) to selected targets was correlated with the magnitude of protein-level change on IMP1 knockdown. The linear regression line is shown with 95% confidence interval (shaded area). Pearson correlation coefficient (r) and p-value are indicated. Key targets are labelled.

**Supplementary Table 1.** CRISPR guide RNA (gRNA) sequences and corresponding PAM motifs. All gRNAs were purchased from Synthego.

**Supplementary Table 2.** RT-qPCR primer sets used in this study, including target gene and forward/reverse primer sequences (5'–3'). Primers were synthesized by Sigma Aldrich.

## Supplementary Figure 1

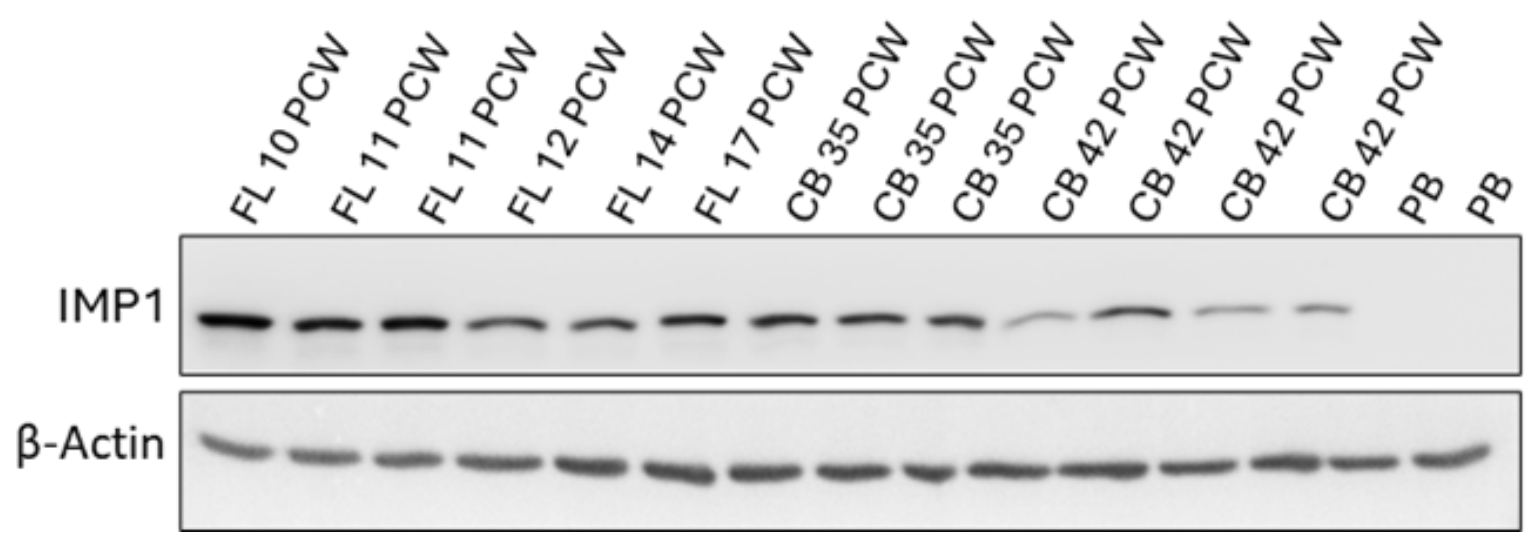

# Supplementary Figure 2

A)

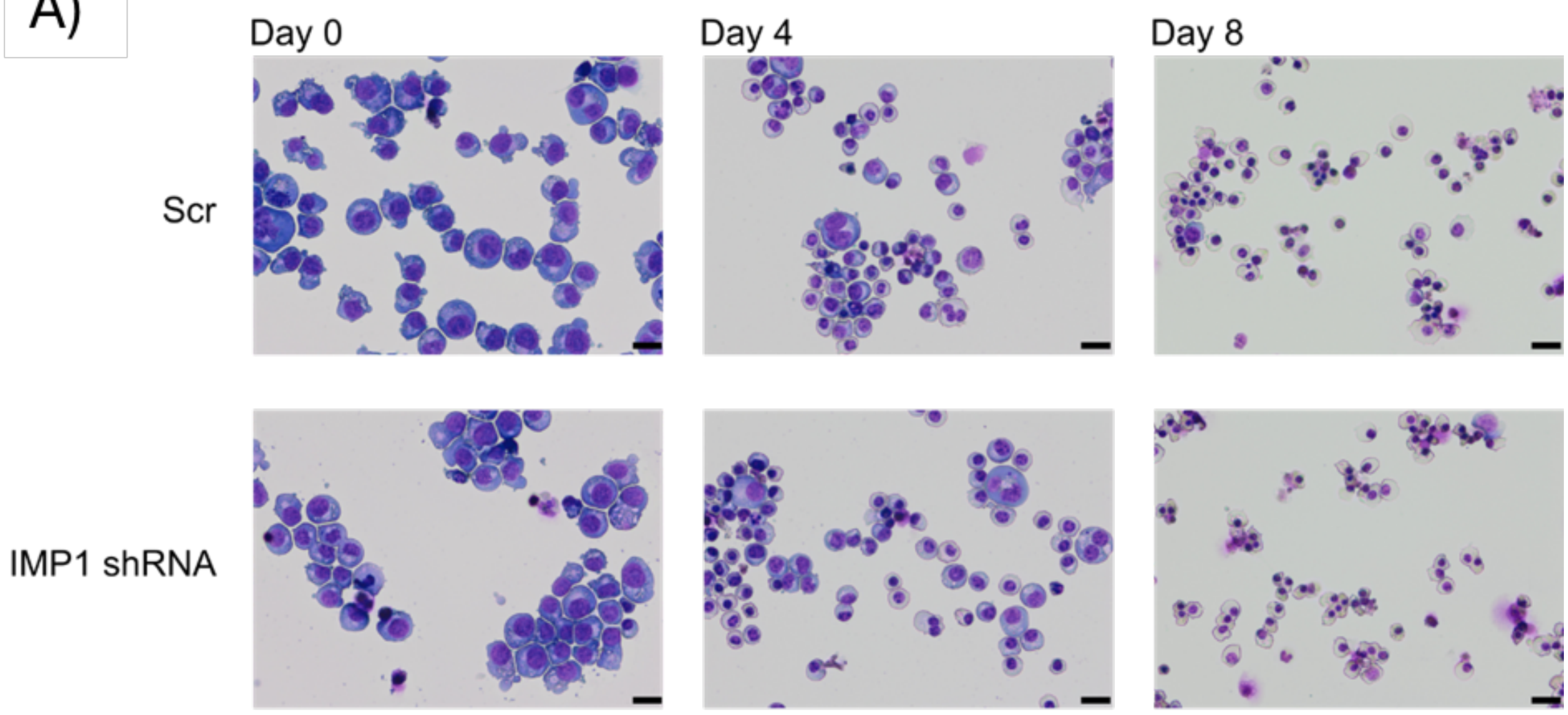


B)

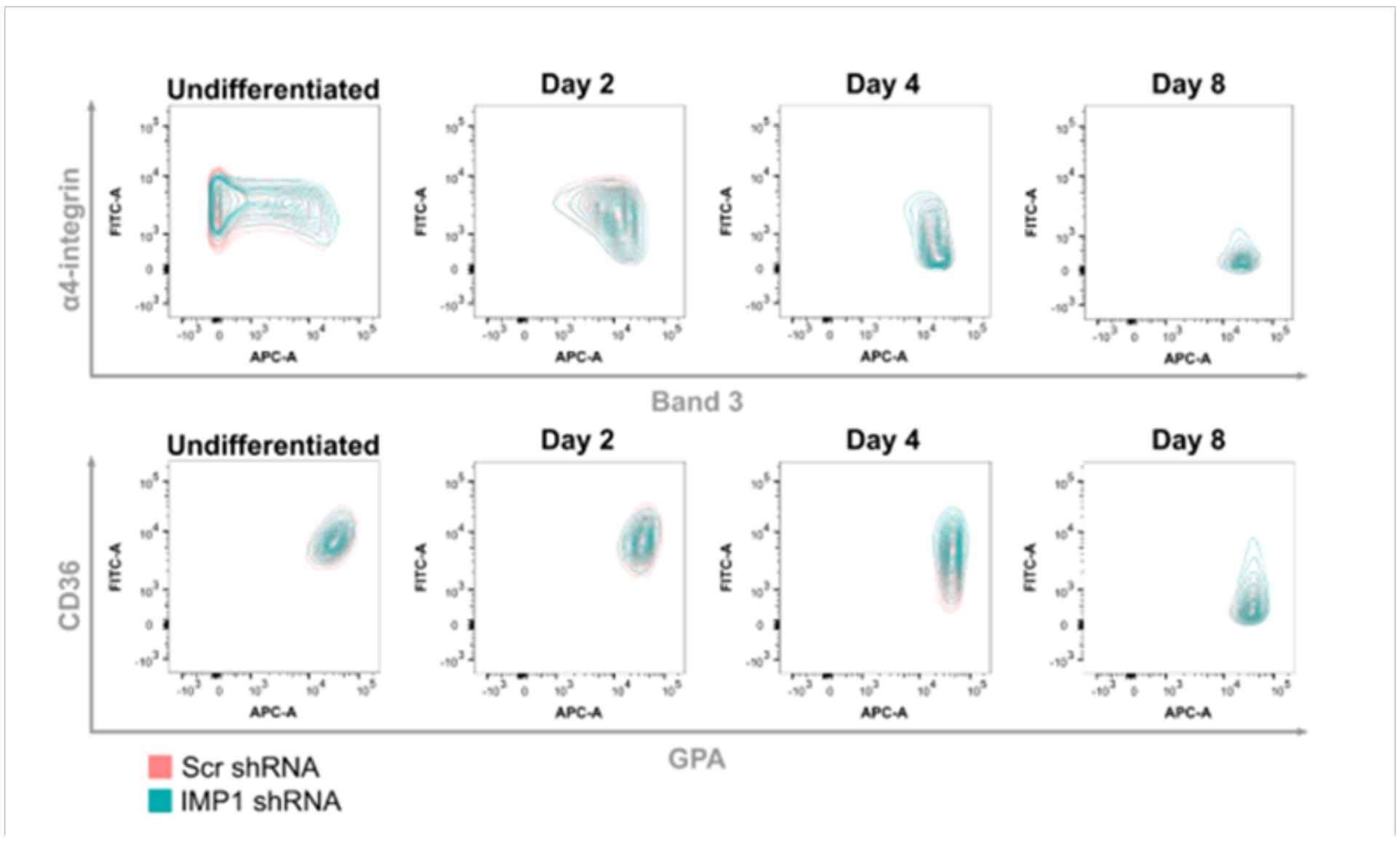

# Supplementary Figure 3

A)

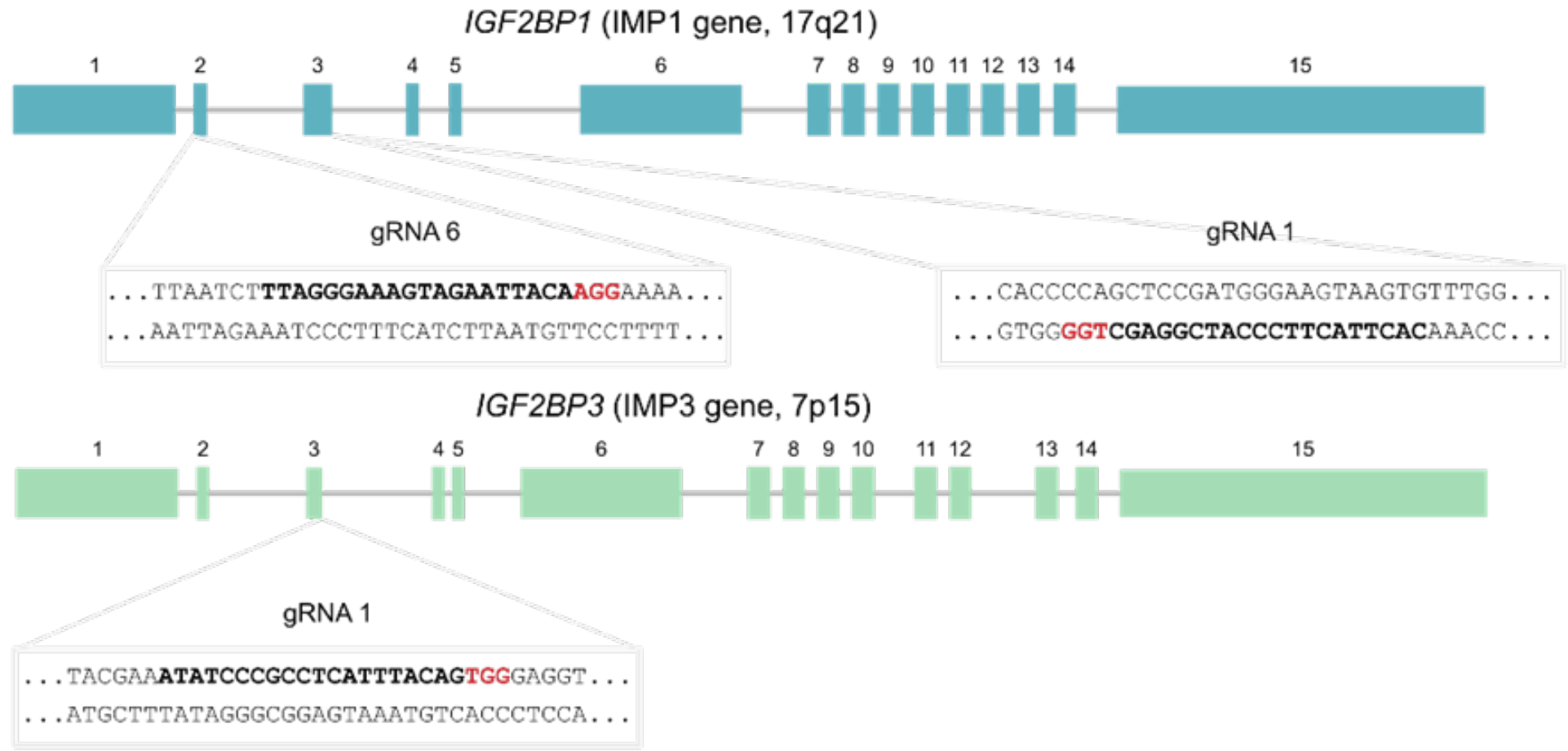


B)

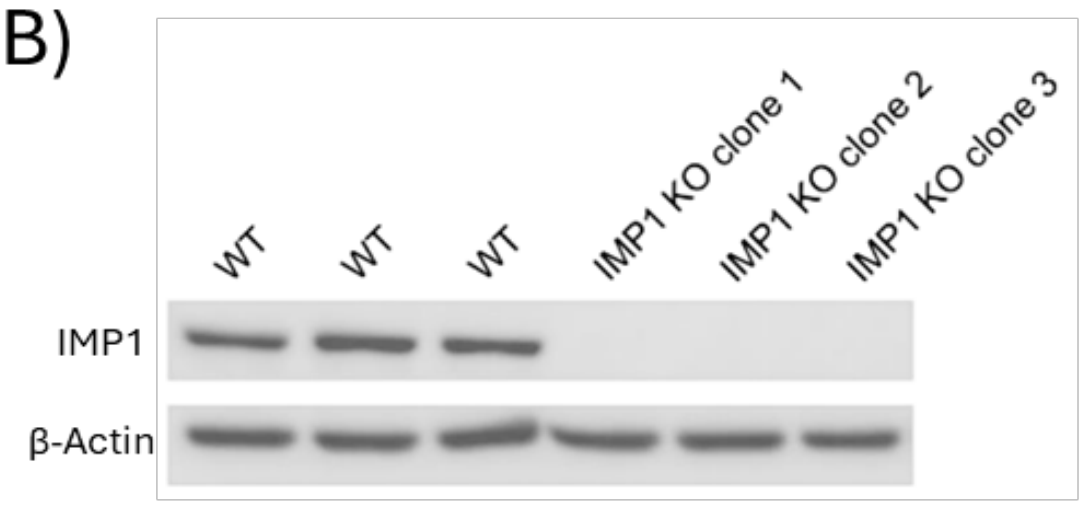


C)

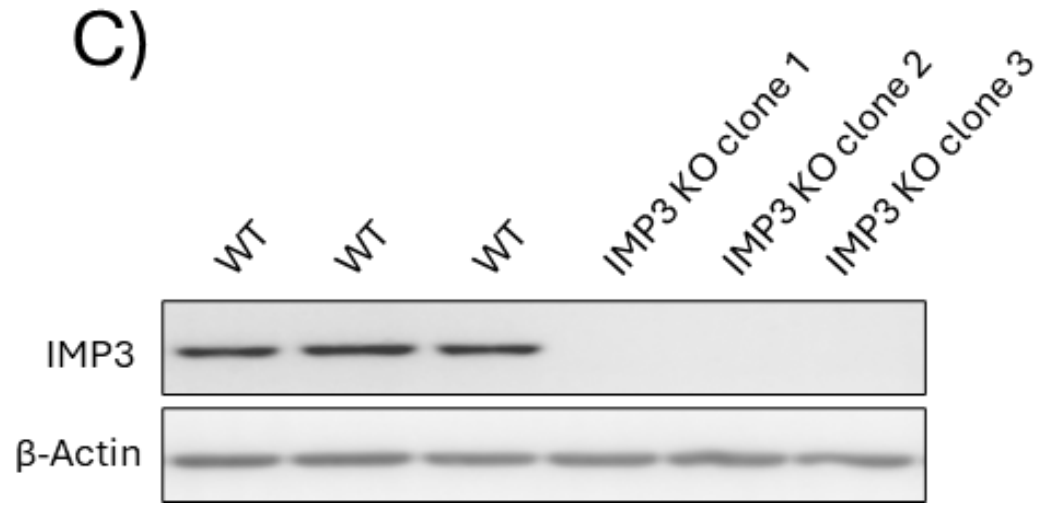

## Supplementary Figure 4

A)

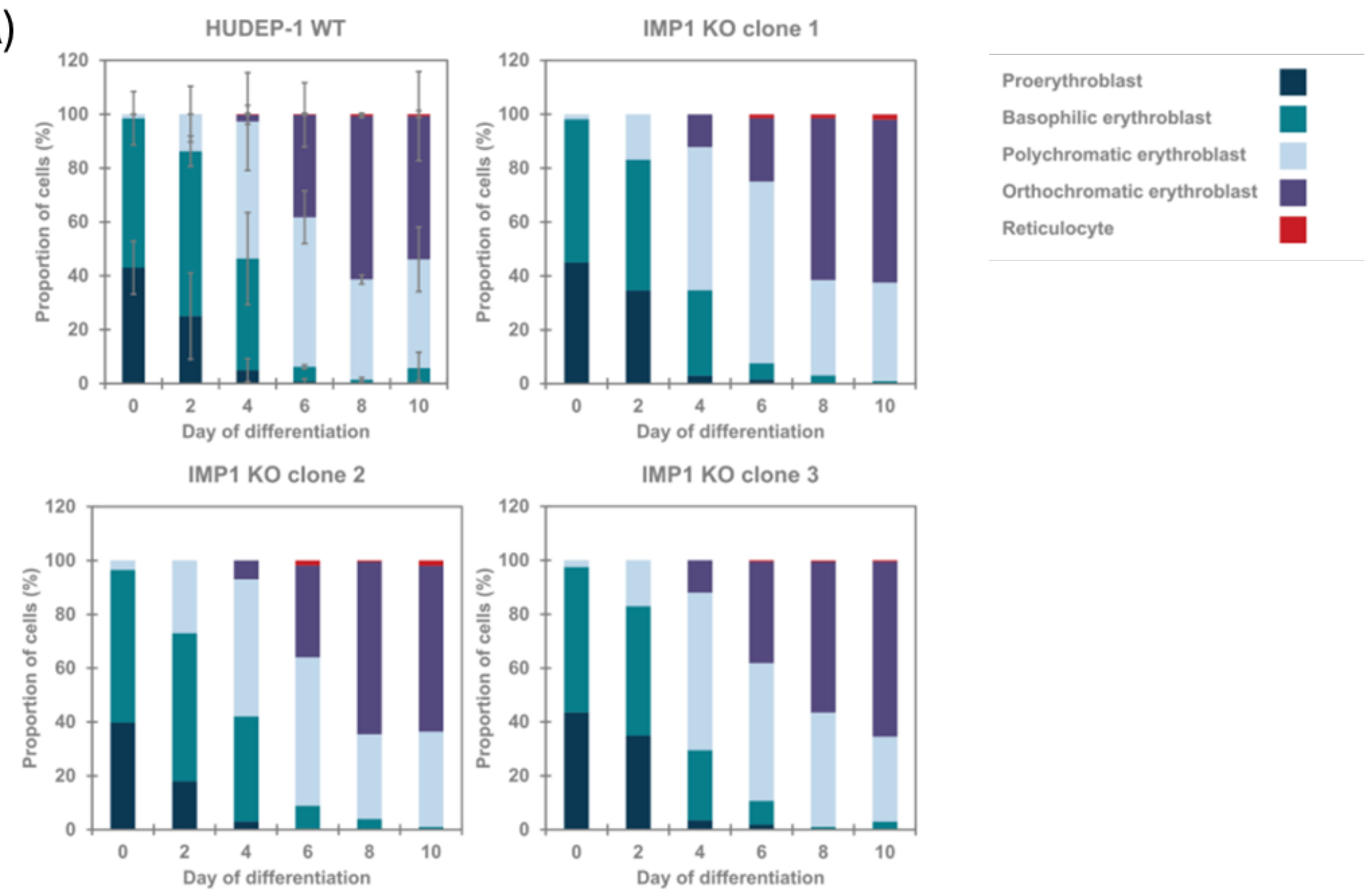

## Supplementary Figure 5

A)

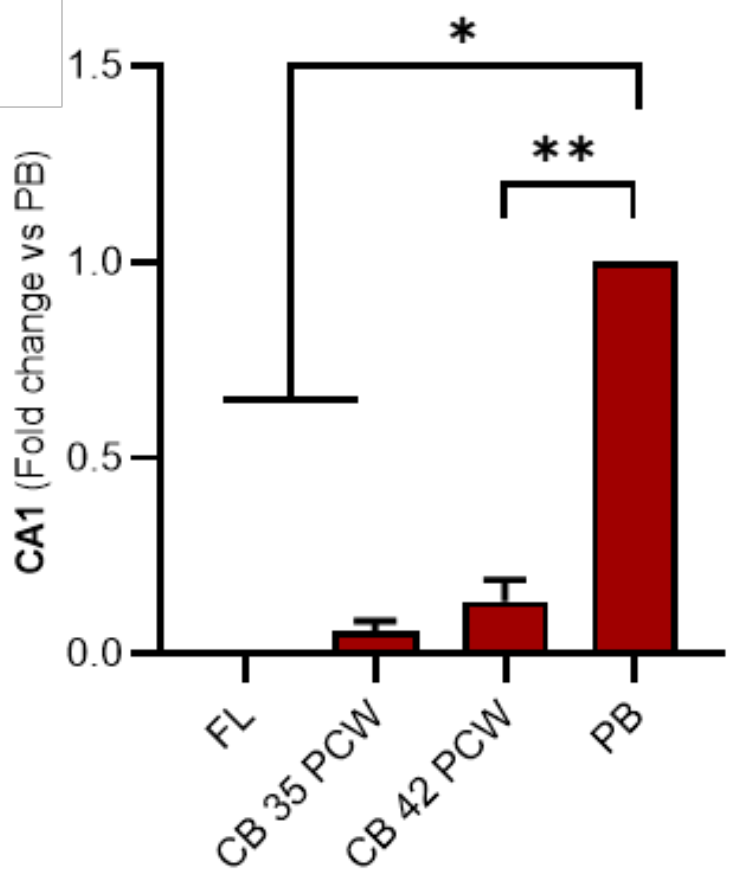


B)

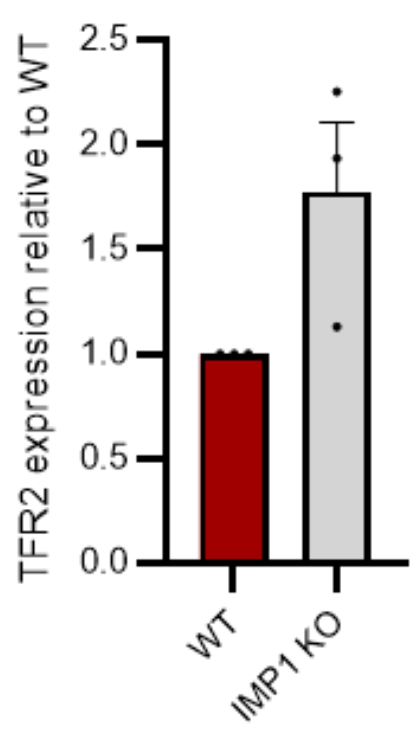


C)

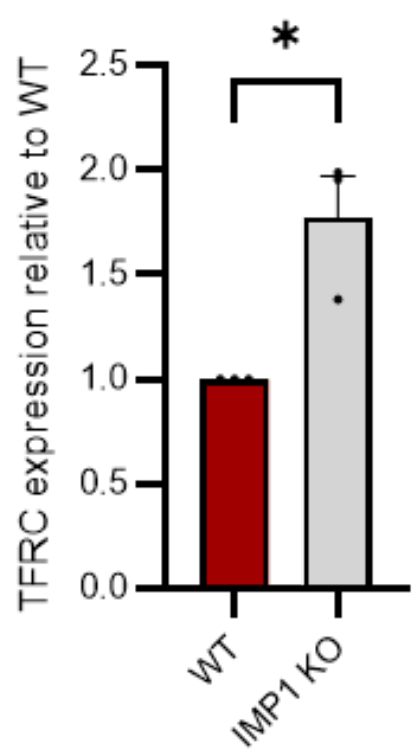

# Supplementary Figure 6

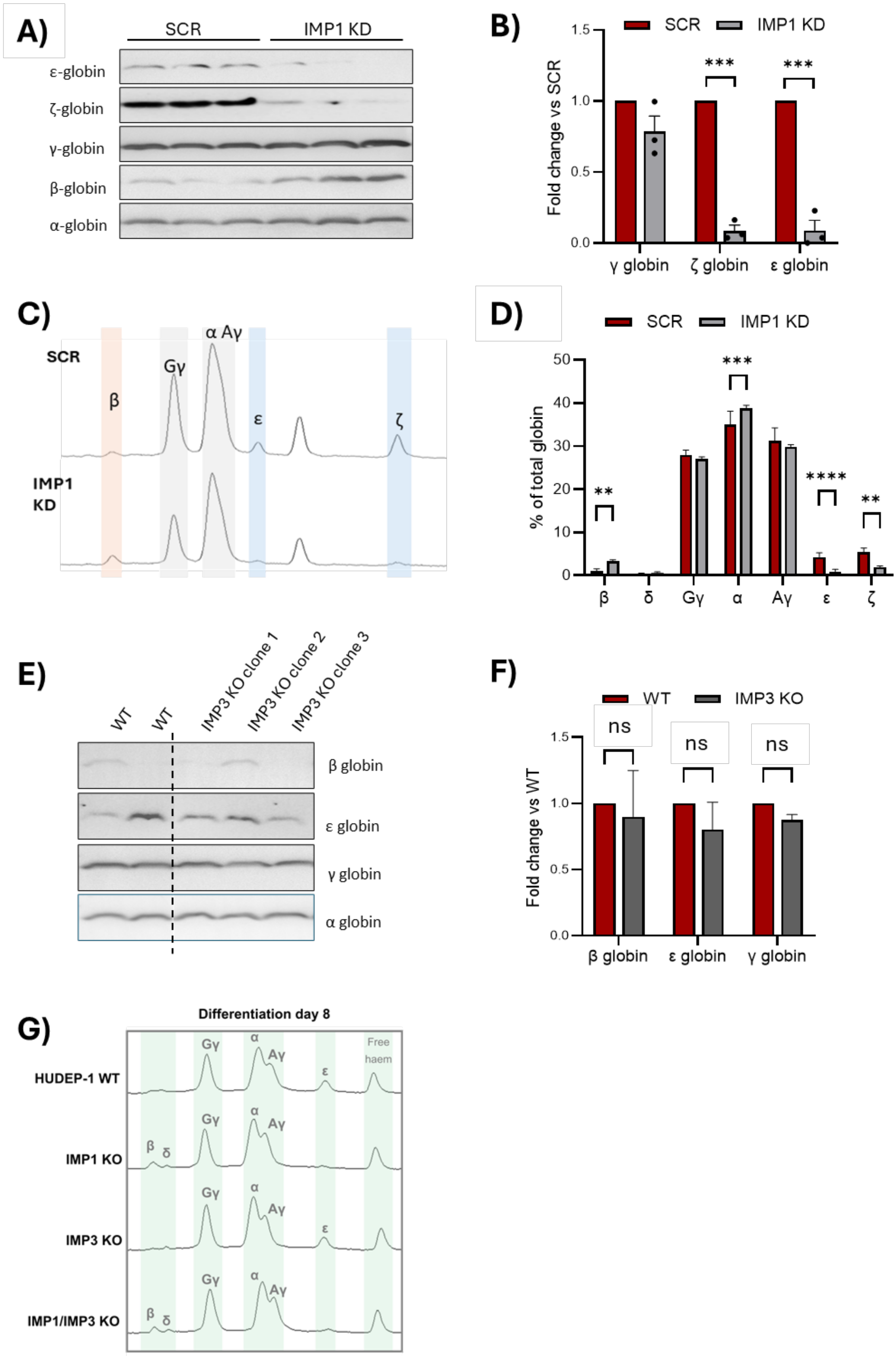

## Supplementary Figure 7

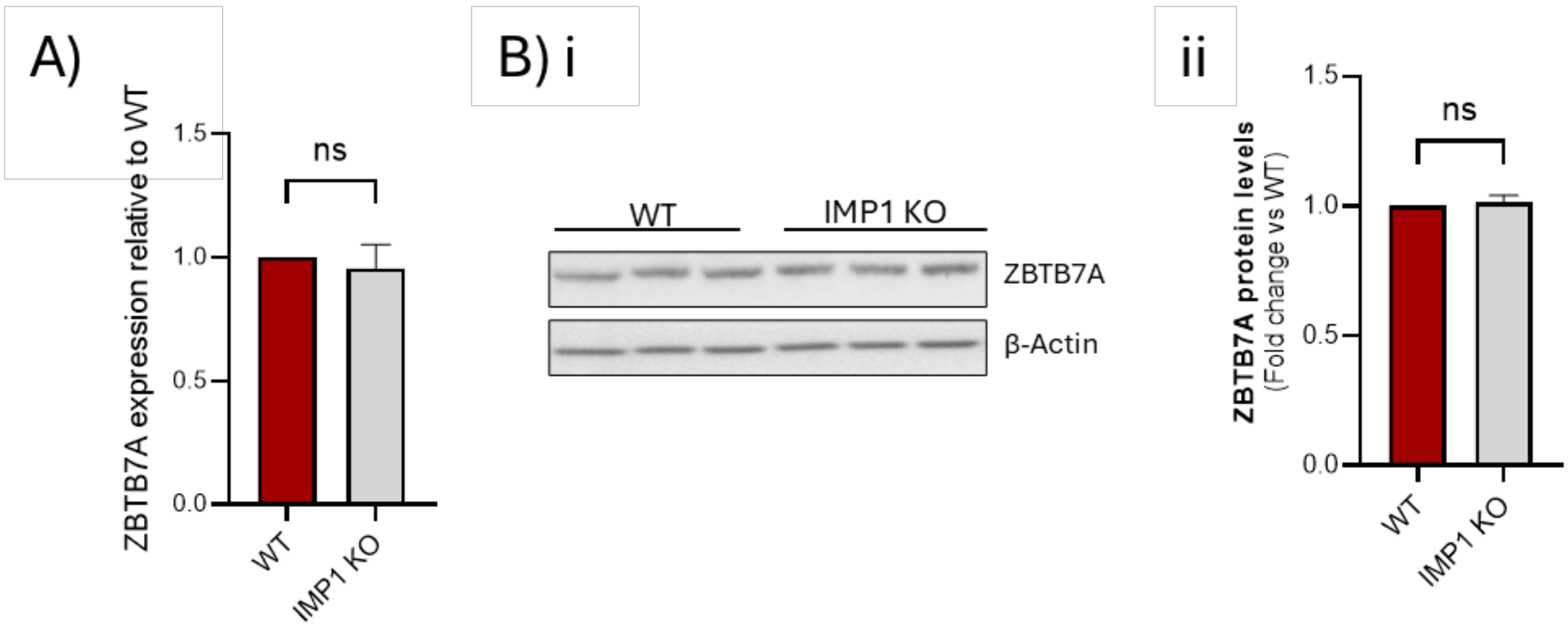


## Supplementary Figure 8

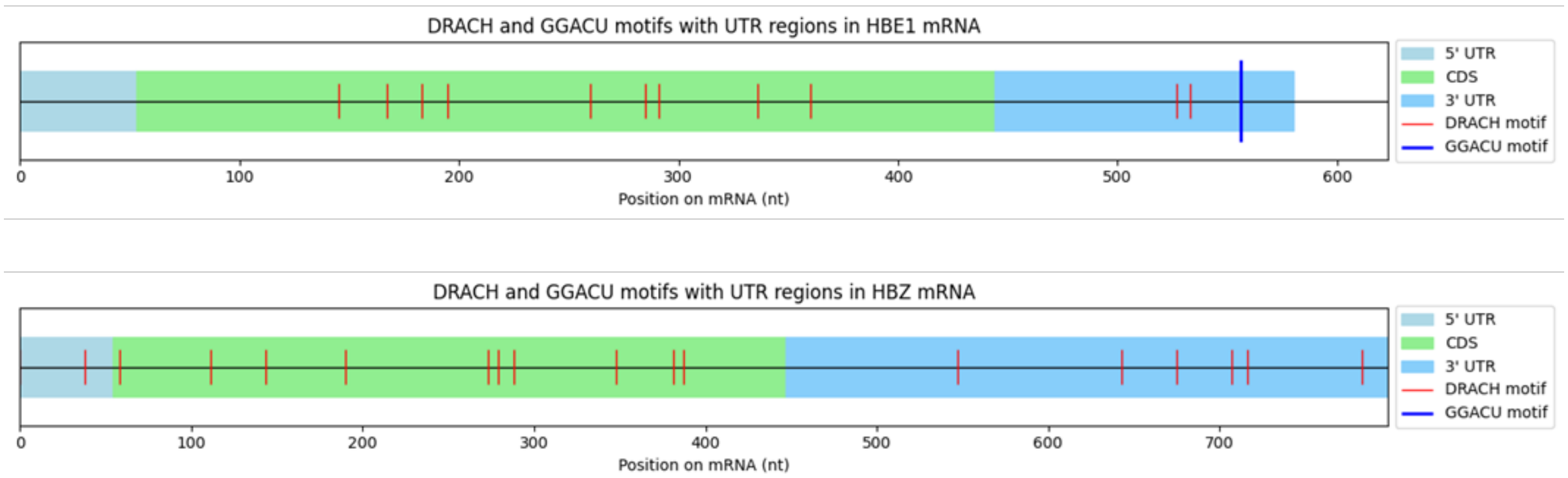


## Supplementary Figure 9

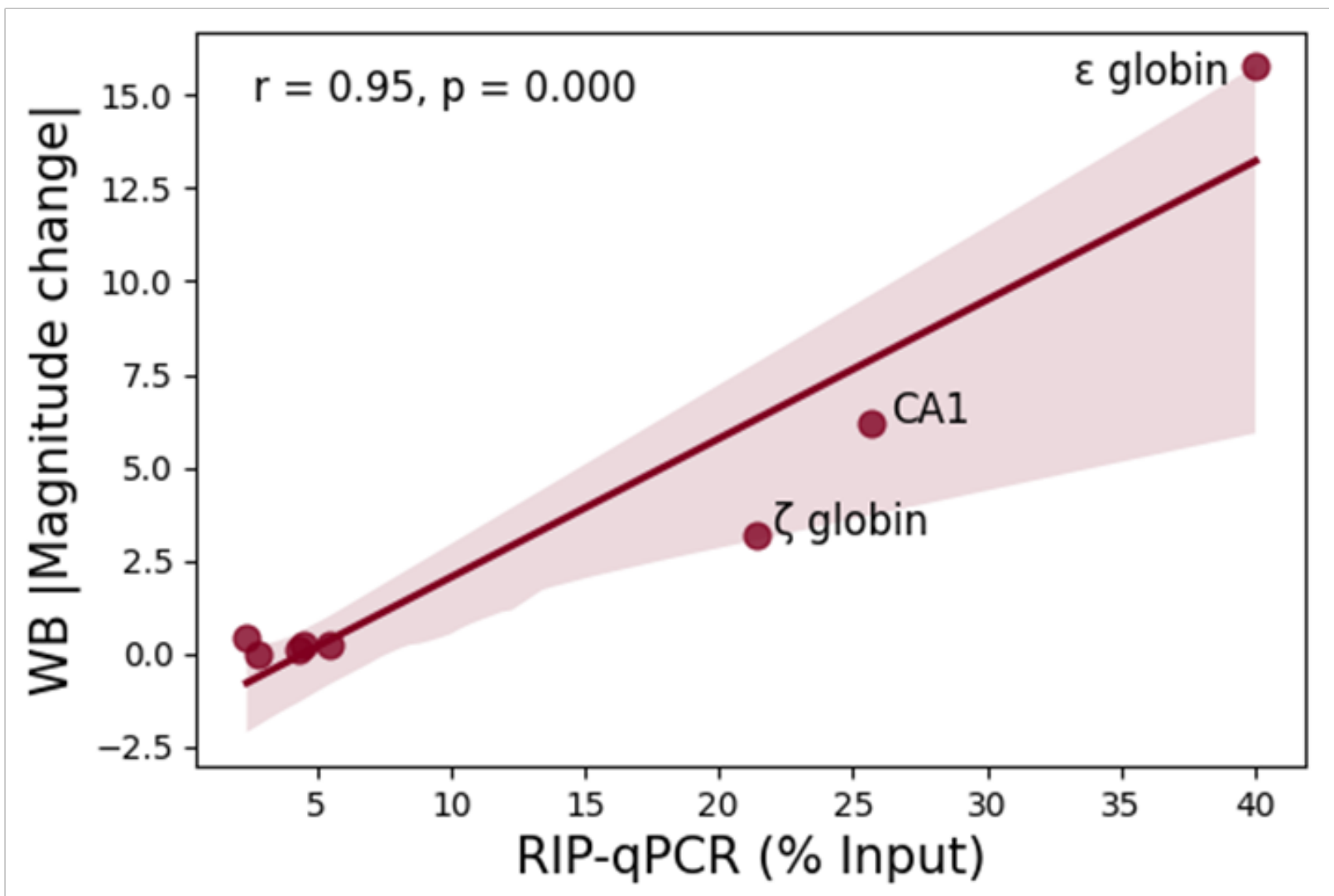

## Supplementary Table 1

| sgRNA name | sgRNA target sequence | PAM |
|---|---|---|
| IMP1 sgRNA 1 | CACTTACTTCCCATCGGAGC | TGG |
| IMP1 sgRNA 6 | TTAGGGAAAGTAGAATTACA | AGG |
| IMP3 sgRNA 1 | ATATCCCGCCTCATTTACAG | TGG |

## Supplementary Table 2

| Target | Forward primer sequence | Reverse primer sequence |
|---|---|---|
| IMP1 | TATAGCTCCTTTATGCAGGCTCC | TGTTTGATGTGCTGCCCCTT |
| IMP3 | TTGAGCACTCGGTCCCAAAA | ACTATCCAGCACCTCCCACT |
| ARID3A | CTGGCGGCTCGGTTTCT | TCAAGCTGAAGGCTGATGGGG |
| BCL11A | CCCGCAGGGTATTTGTAAAG | GAGTGTTCTGTGCGTGTTGC |
| ZBTB7A | GGAGAAGAAGATCCGAGCCA | CAGCTTGTCCTGCCTGGTG |
| CA1 | GATGAAGGTTGGTGAGGCCA | GCTCGTTTGCCCTTGGTTTT |
| CDH1 | GATCTTCAATCCCACCACGTACA | GGTATTGGGGGCATCAGCAT |
| MAPK1 | CGCCGAAGCACCATTCAAG | TCTGAGCCCTTGTCCTGA |
| PABPC | AGCTGTTCCCAACCCTGTAATC | GGATAGTATGCAGCACGGTTCTG |
| TFRC | GAGGACGCGCTAGTGTTCTT | GGCTGAACCGGGTATATGACA |
| TFR2 | TCTATTCCAGAGAGCGCAACA | GAAGTGGGCCAATGTCTCCG |
| RPLP0 | CGTCCTCGTGGAAGTGACAT | TAGTTGGACTTCCAGGTCGC |
| HBE1 | GGGCAGACTCCTCGTTGTT | GCCTTGACCTTGGGGTTG |
| HBZ | CTCCCCTCACCTGACCAAT | GACTGGCCTTGGTAGTGCTC |
| HBG | TGGGTCATTTCACAGAGGAG | TAGACAACCAGGAGCCTTCC |
